# Possible Roles of Calreticulin in Uterine Decidualization and Receptivity in Rats and Humans

**DOI:** 10.3390/ijms221910505

**Published:** 2021-09-29

**Authors:** Mikihiro Yoshie, Kazuya Kusama, Risaka Tanaka, Takanori Okubo, Junya Kojima, Yotaro Takaesu, Keiichi Isaka, Hirotaka Nishi, Kazuhiro Tamura

**Affiliations:** 1 Department of Endocrine Pharmacology, Tokyo University of Pharmacy and Life Sciences, Tokyo 192-0392, Japan; yoshie@toyaku.ac.jp (M.Y.); kusamak@toyaku.ac.jp (K.K.); risaka629@gmail.com (R.T.); t_okubo@yokohama-cu.ac.jp (T.O.); 2 Department of Obstetrics and Gynecology, Tokyo Medical University, Tokyo 160-0023, Japan; kojima_j@tokyo-med.ac.jp (J.K.); isaka@tokyo-med.ac.jp (K.I.); nishih@tokyo-med.ac.jp (H.N.); 3 St. John’s Society Sakuramachi Hospital, Tokyo 184-8511, Japan; hikarinosasuhoue0428@yahoo.co.jp

**Keywords:** calreticulin, decidualization, polypectomy, infertility

## Abstract

Previous in vitro studies have suggested that calreticulin (CALR), which is responsible for the folding and quality control of glycoproteins, may be associated with decidualization. However, its precise role in regulating decidualization has not been explored in vivo. Here, we used pregnant rat models to examine endometrial CALR expression during the peri-implantation period. We also examined whether polypectomy, a procedure that could ameliorate infertility, alters the endometrial expression levels of CALR and several implantation factors in women diagnosed as infertile. In rats, uterine CALR was expressed at a high level at the implantation site, and a marked increase in CALR expression was observed in decidual cells of normal pregnancy. In addition, endometrial CALR expression was enhanced by either administration of estradiol-17β in the delayed implantation rat model or the artificial induction of decidualization in the pseudopregnant rat. In cultured stromal cells, siRNA-mediated silencing of CALR inhibited the decidual stimulus-induced expression of prolactin, decidual/trophoblast prolactin-related protein, and connexin 43. In humans, the endometrial expression levels of the mRNAs encoding CALR and the implantation-related factor insulin-like growth factor binding protein (IGFBP)-7 tended to increase after polypectomy. The strongest positive correlation between expression levels before polypectomy was observed for IGFBP-7 and CALR, and the strength of this correlation increased after the surgery. Thus, endometrial CALR may play a role in the formation of decidua, and the polypectomy of infertile patients may result in the co-operative expression of endometrial factors, including CALR, that could enhance endometrial receptivity.

## 1. Introduction

Uterine endometrial stromal cells (ESCs) differentiate into decidual cells during the late secretory phase of the menstrual cycle to prepare for implantation [1]. The successful establishment of implantation and pregnancy requires the appropriate growth of blastocysts and the concomitant acquisition of endometrial receptivity, including stromal cell decidualization [1,2]. However, the biochemical mechanisms involved in ESC decidualization have yet to be elucidated conclusively. The elevation of intracellular cyclic adenosine monophosphate (cAMP) levels triggered by ovarian steroids plays a crucial role in successful decidualization [3]. In addition, previous reports have demonstrated that the regulation of calreticulin (CALR) by a cAMP mediator, named an exchange protein, through direct activation by cAMP-2 (EPAC2) may be involved in the decidualization of human ESCs [4,5]. CALR is a highly conserved chaperon protein localized mainly in the endoplasmic reticulum (ER), where it functions as a chaperone protein involved in protein folding, maturation, and trafficking [6]. In addition, CALR regulates the quality of newly synthesized glycoproteins together with calnexin and ERp57 and controls the homeostasis of intracellular Ca^2+^ levels [6,7,8]. CALR plays a role in many aspects of cellular functions, both inside and outside the ER. For example, CALR promotes the uptake of tumor cells by enhancing phagocytosis in macrophages, and the mutation of CALR alters the proliferation of hematopoietic cells [9].

Several reports highlight the importance of CALR in female reproduction. The study using CALR knockout mice demonstrated that CALR may play an indispensable role in the development of a cumulus oocyte complex by regulating the maturation of GDF9 and BMP15 proteins [10]. Furthermore, previous studies have shown that CALR is expressed in the uterus of primates, including humans [11], and mice [12], and further still, this chaperon may mediate maturation of the endometrial glandular and luminal epithelium, leading to the up-regulation of the leukemia inhibitory factor (LIF) and prostaglandin G/H synthase 2 (PTGS2/COX2), both of which are related to endometrial receptivity [13]. Thus, impaired CALR function leads to female infertility. However, the precise mechanism by which CALR regulates uterine functions has not been characterized.

Intriguingly, approximately 8% of patients with endometrial abnormalities undergoing in vitro fertilization possess endometrial polyps [14]. Polypectomy and myomectomy in patients with uterine polyps and fibroids, respectively, seem to improve the rate of pregnancy and could be an effective therapeutic measure [15]. Numerous reports have identified the endometrial molecules that are involved in embryo receptivity and decidualization, including the insulin-like growth factor binding protein 1 (IGFBP1), IGFBP7, and PTGS2 [1,2]. However, to our knowledge, no studies have evaluated the molecular events associated with changes in the receptivity of the endometrium and embryo implantation in the same patients before and after polypectomy. We propose that structural and unknown functional abnormalities of endometrial receptivity induced by these benign tumors may be ameliorated by surgical removal of the lesions.

In the present study, to explore the relationship between endometrial CALR and decidualization in vivo, CALR expression was examined in a rat model of implantation and decidualization. In addition, the expression levels of endometrial CALR and other implantation-related factors were compared in the endometrium of infertile patients before and after polypectomy.

## 2. Results

### 2.1. CALR Is Spatiotemporally Expressed in the Peri-Implantation Uterus in Rats

**Figure 1:**
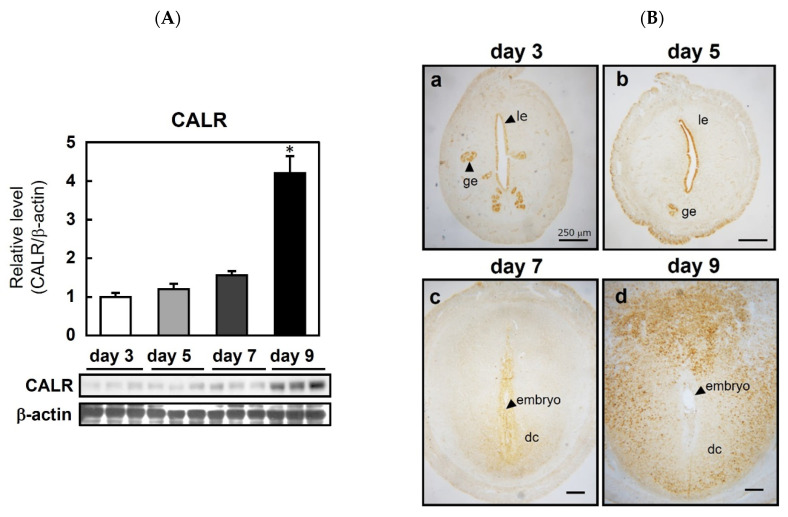
Expression of calreticulin (CALR) in the peri-implantation uterus of rats. Immunoblot analysis of CALR in the rat uterus on the indicated days of pregnancy (**A**). CALR levels were normalized to those of β-actin. Data represent the mean ± SEM (*n* = 3). * *p* < 0.01 versus day 3. Immunolocalization of CALR in the pregnant rat uterus (**B**); le, luminal epithelial cells; ge, glandular epithelial cells; s, stromal cells; dc, decidual cells. Scale bars = 250 μm.

To determine the cell-specific expression pattern of CALR, uterine sections from pregnant rats were examined before and after embryo implantation (Figure 1). In rats, a nidatory surge of estrogen on the afternoon of day 5 of pregnancy triggers implantation on day 6, thereby allowing the blastocyst to attach to the epithelial layers of the receptive uterus. Decidualization is initiated immediately in the endometrial cells adjacent to the implantation sites during blastocyst attachment and invasion [16]. On day 9 of pregnancy, approximately 3 days after embryo attachment, the level of the uterine CALR protein in the pregnant rats was significantly higher than that on days 3, 5, and 7 (Figure 1A). The CALR protein was localized in the glandular and luminal epithelium on days 3 (Figure 1(Ba)) and 5 (Figure 1(Bb)); however, on day 7 (i.e., after implantation), localization was observed in decidual cells surrounding the embryo (Figure 1(Bc)), and the intensity of CALR staining on day 9 was more prominent than that on day 7 (Figure 1(Bd)).

### 2.2. CALR Expression in an Artificial Implantation and Decidualization Rat Model

**Figure 2:**
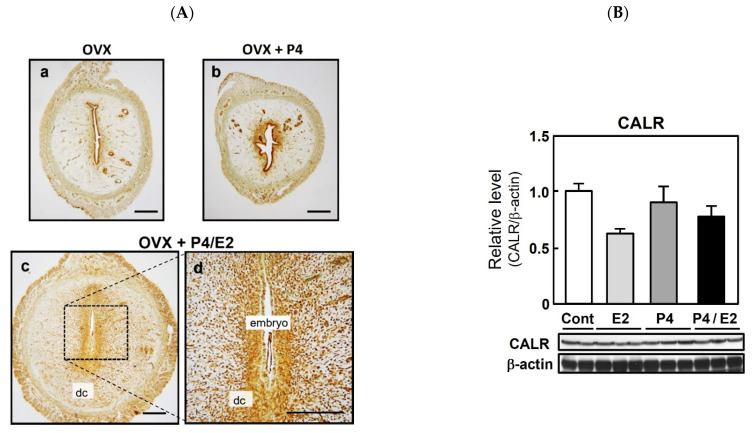
Expression of calreticulin (CALR) in the delayed implantation model and effects of ovarian steroids on uterine CALR expression in rats. Pregnant rats were ovariectomized (OVX) on day 4 of pregnancy and injected with or without progesterone (P4, 3 mg/day) on days 4 to 9 to delay implantation (**Aa**,**Ab**). *n* = 3. To initiate implantation in some P4-primed rats, estradiol-17β (E2, 500 ng) was injected on day 8 of pregnancy (**Ac**,**Ad**). *n* = 3. Uterine sections were prepared 48 h after E2 injection and CALR was detected by immunostaining; dc, decidual cells. Scale bars = 250 μm. Ovariectomized rats were given injections of P4 (3 mg) daily for 3 days and/or a single injection of E2 (500 ng) on the third day (**B**). Control (Cont) rats received neither steroid. Uteri were collected 24 h after E2 injection and/or the final P4 injection. CALR was detected in uterine lysates via immunoblotting and its expression level was normalized to that of β-actin. Data represent the mean ± SEM (*n* = 3).

To determine whether CALR expression is associated with the induction of implantation and regulated by ovarian hormones, immunohistochemical analyses were performed using the P4-treated delayed implantation model (Figure 2A). Similar to its localization on days 3 and 5 of normal pregnancy, CALR was limited to the glandular and luminal epithelium in both control ovariectomized rats (Figure 2(Aa)) and ovariectomized rats injected with P4 to delay implantation (3 mg/day from days 4 to 9 of pregnancy, Figure 2(Ab)). However, the injection of the P4-treated rats with E2 (500 ng) on day 8 of pregnancy, which mimics the nidatory surge of estrogen to initiate implantation, enhanced the expression of CALR in both decidual cells and epithelial cells (Figure 2(Ac,Ad)). The same doses of ovarian steroids (E2 and/or P4) had no significant effect on CALR expression in the uterus of ovariectomized rats (Figure 2B).

**Figure 3:**
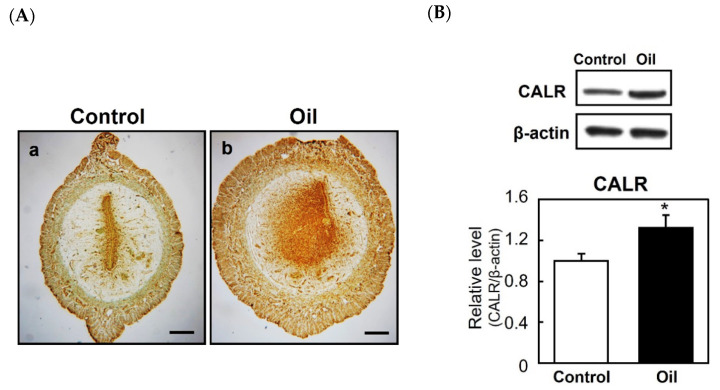
Expression of calreticulin (CALR) in the artificially induced decidua. Female rats were mated with vasectomized males and sesame oil (100 μL) was infused into the lumen of one uterine horn on day 5 of pseudopregnancy to induce artificial decidualization. The uteri were dissected 48 h after the infusion. Immunostaining of CALR in control (**Aa**) or oil injected horn (**Ab**). Scale bars = 250 μm. Immunoblot analyses of CALR expression in the uteri of control or those injected with sesame oil (**B**). The graph shows the relative level of CALR normalized to that of β-actin. Data represent the mean ± SEM (*n* = 3). * *p* < 0.05.

To examine the relationship between decidualization and CALR expression further, pseudopregnant rats were given an artificial stimulus (sesame oil infusion) to induce decidualization. Immunostaining of the uterus revealed that the injection of sesame oil into the uterine lumen stimulated the expression of CALR in the decidualizing stroma (Figure 3(Aa,Ab)). In addition, an immunoblot analysis showed that the level of CALR in the uterine horn was increased significantly following the injection of sesame oil (Figure 3B), indicating an enhanced CALR expression during the development of the deciduoma in the absence of blastocysts. Overall, these results suggest that an elevation of endometrial stromal CALR is associated with decidualization.

### 2.3. Knocking down CALR Reduces the Expression of Decidual Markers in Cultured Rat Stromal Cells

**Figure 4:**
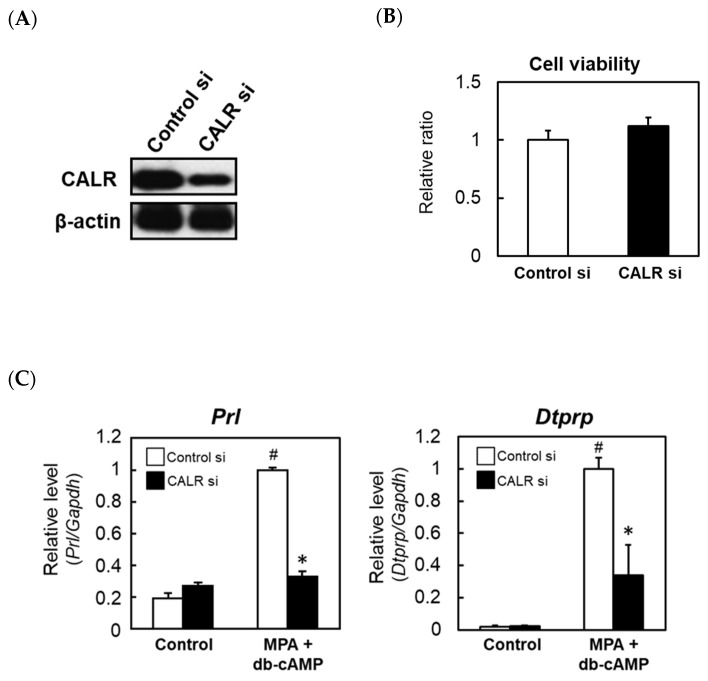

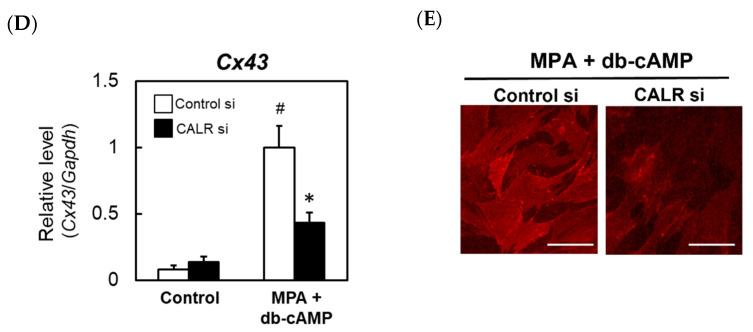
The effects of knocking down calreticulin (CALR) on the expression levels of decidual markers in an in vitro rat decidualization model. Rat ESCs were treated for 24 h with a non-targeting control (Cont) or CALR-specific siRNA and then cultured for an additional 24 h. Immunoblot analysis of CALR expression (**A**). The viabilities of rat ESCs transfected with a non-targeting control or CALR-specific siRNA were assessed using the WST-8 assay (**B**). Real-time RT-PCR analyses of the Prl (**C**), Dtprp (**C**), and Cx43 (**D**) mRNAs in rat ESCs transfected with a non-targeting control or CALR-specific siRNA. At 48 h after the siRNA transfection, cells were co-treated with MPA (100 nM) and db-cAMP (500 μM) for a further 48 h. Total RNA was then extracted from the cells and subjected to real-time RT-PCR. The expression level of Gapdh was used as an internal control. Data represent the mean ± SEM of three independent experiments. # *p* < 0.01 versus Control; * *p* < 0.01 versus Control siRNA. Immunocytochemical analysis of Cx43 in rat ESCs transfected with a non-targeting control or CALR-specific siRNA (**E**). Scale bar = 100 mm.

To explore the function of increased CALR expression in decidualizing cells, rat ESCs were transfected with a CALR-specific siRNA. Immunoblotting confirmed that transfection with the specific siRNA reduced CALR protein levels in cultured stromal cells (Figure 4A). Cell viability was not affected by the knockdown of CALR (Figure 4B). Enhanced expression levels of prolactin (PRL) and decidual/trophoblast prolactin-related protein (DTPRP) are markers of decidualization in rats; therefore, we examined the effects of knocking down CALR on Prl and Dtprp expression. In rat ESCs transfected with a non-specific control siRNA, decidual stimulation by co-treatment with medroxyprogesterone (MPA) and dibutyrly (db)-cAMP increased the expression levels of the Prl and Dtprp mRNAs significantly (Figure 4C). However, the knockdown of CALR attenuated these responses significantly (Figure 4C).

Cx43, a membrane-spanning connexin protein that forms gap junctions, is expressed in the decidual zone, and both Cx43 and gap junctions are required for ESC differentiation [16]. Therefore, we examined whether Cx43 is regulated by CALR. The treatment of control rat ESCs with MPA and db-cAMP increased Cx43 mRNA expression significantly, and this increase was attenuated by the siRNA-mediated knockdown of CALR (Figure 4D). A reduced expression of Cx43 protein in CALR knockdown cells was also confirmed by an immunocytochemical analysis (Figure 4E).

### 2.4. The Effect of Endometrial Polypectomy on Implantation-Related Factors in Infertile Patients

**Figure 5:**
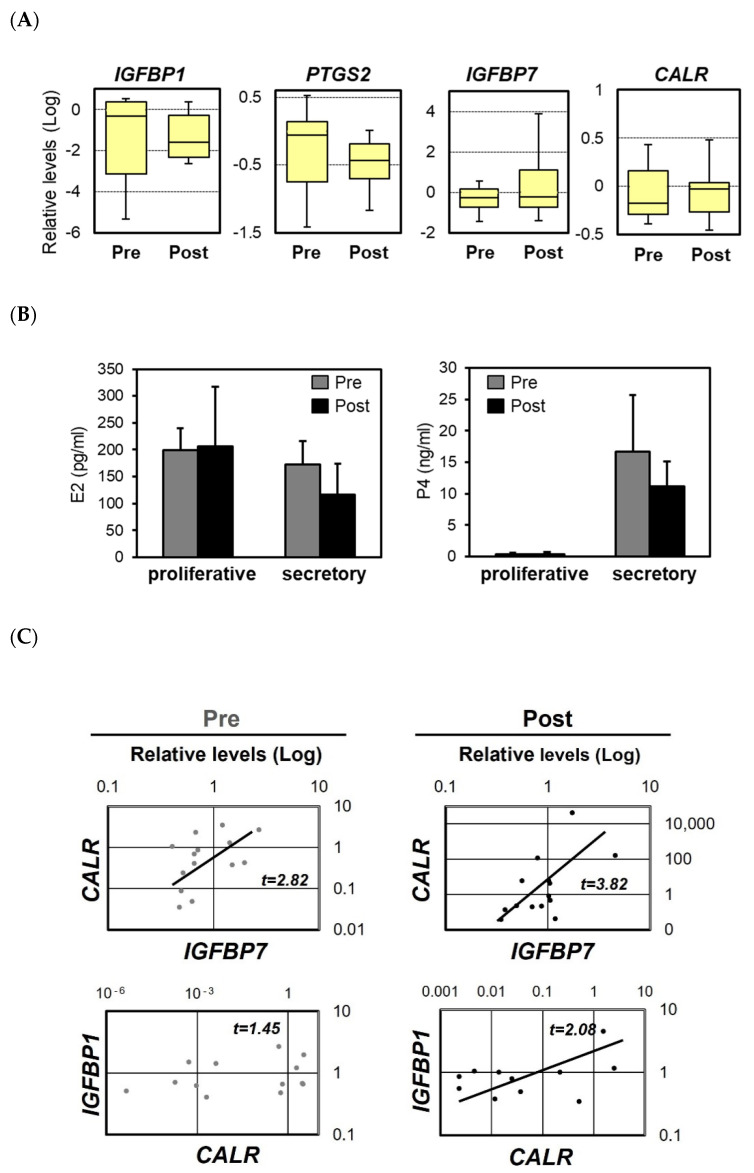
The expression levels of the mRNAs encoding insulin-like growth factor 1 (IGFBP1), prostaglandin G/H synthase 2 (PTGS2), IGFBP7, and calreticulin (CALR) in infertile patients before and after polypectomy. The expression levels of the *IGFBP1*, *PTGS2, IGFBP7,* and *CALR* mRNAs in the human endometrium before and after polypectomy (**A**). The scales indicate the relative expression levels of each mRNA. The bottom and top of each box indicate the 25th and 75th percentiles, respectively, and the band in the middle of the box indicates the median value (of 27 patients). The concentrations of estradiol-17β (E2) and progesterone (P4) in serum during the proliferative and secretory phases of the uterine cycle, as measured using electrochemiluminescence immunoassay (**B**). Samples were collected before and after polypectomy. Data represent the mean ± SEM of 27 patients. Correlations between the mRNA expression levels of *CALR* and *IGFBP1* or *IGFBP7* in the endometrium of infertile patients before and after polypectomy (**C**). Relative mRNA expression levels were detected by real-time RT-PCR.

Endometrial polypectomy reportedly improves the rate of pregnancy in women previously diagnosed as infertile [15], although the evidence from clinical studies is scarce and conflicting [17,18]. To determine whether polypectomy influences the expression of implantation-related factors, we examined the effects of excision on the expression levels of the *IGFBP1*, *IGFBP7*, *PTGS2*, and *CALR* mRNAs in 27 patients with endometrial polyps. Pre-polypectomy samples were collected from normal areas of the endometrium during surgery and post-polypectomy samples were collected from the same patients 1–2 months later. The expression levels of *IGFBP1* and *PTGS2* tended to decrease after the surgery, whereas those of *IGFBP7* and *CALR* increased slightly (Figure 5A). Polypectomy had no significant effect on the serum levels of E2 and P4 in the luteal phase (Figure 5B). Among all factors examined, the strongest positive correlation before polypectomy was observed for *IGFBP7* and *CALR*, and the strength of this correlation increased further after excision (Figure 5C).

## 3. Discussion

The transformation of ESCs into decidual cells is a unique functional change required for embryo implantation during the secretory phase [1,2,3]. Decidualization is characterized by the up-regulation of genes encoding proteins such as decidual PRL and IGFBP1 and the morphological change from a fibroblastic to an enlarged epithelioid shape. Impaired decidualization causes infertility and recurrent miscarriage [19,20]. Here, we found that CALR is expressed preferentially at the site of blastocyst implantation, and its expression is induced during the progression of decidualization in rats. In addition, we found that the knockdown of CALR expression suppressed the decidualization of endometrial cells. There has been little evidence showing the specific expression of CALR in human decidual tissues, but we have demonstrated previously that the knockdown of CALR inhibits the decidualization of cultured human ESCs [4]. Furthermore, CALR expression is reportedly higher in the stromal region of the endometrial functionalis in pregnant primate bonnet monkeys compared with non-pregnant animals [11]. In a previous mouse study, CALR expression in the stroma of pregnant mice was reportedly higher in the implantation pole endometrium than in non-implantation tissues [12]. In the same study, CALR expression in the mouse endometrium increased around the time of implantation, and implantation was blocked by the injection of an antisense CALR oligodeoxynucleotide into the uterine horn during early pregnancy [12]. A similar experiment using stable and specific CALR siRNA would prove the role of CALR in the endometrial receptivity in rats. These reports above suggest a close relationship between stromal CALR expression and implantation and therefore support our present findings.

Our previous study using human endometrial glandular epithelial cells showed that EPAC2-directed CALR may be critical for the expression of LIF and for the PTGS2-mediated production of prostaglandin E2 (PGE2) [13]. In that study, an intense expression of uterine CALR was observed in epithelial cells of the rat endometrium just before implantation, suggesting that CALR might be associated with epithelial preparation for implantation in rats. In our current study, the silencing of CALR expression inhibited the MPA/db-cAMP-induced decidual expression of PRL and DTPRP in cultured ESCs. The mechanism by which CALR regulates decidualization is probably linked to the signaling pathway governed by cAMP in the endometrial stroma, which is similar to the situation in epithelial cells.

Cx43, a component of gap junctions that is expressed ubiquitously, enables intercellular communication to control several biological functions, including cell proliferation and inflammation [21]. A knockdown of Cx43 in uterine stromal cells causes abnormal decidual angiogenesis via the disruption of gap junctions and induction of decidual dysfunction [16]. In our current study, the knockdown of CALR attenuated the decidual stimulation-induced up-regulation of Cx43 in rat ESCs. This finding may imply that CALR-mediated decidual signaling affects the formation of gap junctions, which is essential for stromal differentiation.

CALR regulates the storage and release of Ca^2+^ in the ER and maintains the homeostasis of intracellular Ca^2+^ levels. Previous studies have shown that Ca^2+^ may play a critical role in reproductive physiopathologies involving pregnancy complications and impaired implantation and decidualization [22,23,24]. In addition, blocking Ca^2+^ mobilization can alter cAMP signaling, resulting in the up-regulation of PRL and IGFBP-1 levels [24]. Therefore, it is possible that the knockdown of CALR could inhibit decidualization by impairing Ca^2+^ homeostasis. In addition, several transcription factors, particularly HOXA10, C/EBPβ, and FOXO1, are essential for decidualization in humans [1]. HOXA10 may be involved in endometrial development and uterine receptivity for blastocysts because HOXA10-deficient mice display infertility due to implantation and decidualization failure [25]. Notably, CALR expression is impaired in endometrial stroma cells of HOXA10-knockout mice [26]. Future evaluation of the precise interactions between Ca^2+^ mobilization, cell adhesion molecules, and transcriptional factors is required to delineate the function of CALR in decidualization. A recent study showed that the induction of ER stress stimulates extracellular CALR release and alters the localization of the cell adhesion protein E-cadherin to prevent forskolin-induced fusion of trophoblasts [27]. Excess ER stress in ESCs during decidualization might initiate the release of CALR from the cytoplasm. It is possible that extracellular CALR contributes to endometrial functions. Thus, the potential role of extracellular CALR might explain various pathophysiological functions in the endometrium.

IGFBP7 expression is up-regulated in stromal cells during the receptive phase of the menstrual cycle [28], which is a process that may play a role in decidualization of the endometrial stroma [29,30]. In our current study, we found that the expression levels of IGFBP7 and CALR tended to increase after endometrial polypectomy, and a higher correlation between IGFBP7 and CALR expression was observed after the surgery. A positive correlation was also found between IGFBP1 and CALR after polypectomy. These observations suggest that CALR expression may be coordinated with IGFBP7 and IGFBP1 expression during the receptive phase and that polypectomy may restore the coordinated expression of these implantation factors.

In conclusion, we have demonstrated that endometrial CALR may play a role in the formation of decidua and that polypectomy of infertile patients may result in the co-operative expression of endometrial factors associated with endometrial receptivity, including CALR.

## 4. Materials and Methods

### 4.1. Chemicals and Antibodies

Progesterone (P4, P0130), estradiol-17β (E2, E8875), medroxyprogesterone (MPA, M1629), and the cAMP analog dibutyrly (db)-cAMP (D0260) were purchased from Sigma-Aldrich (St. Louis, MO, USA). Antibodies against CALR (#2891) and Cx43 (#610061) were obtained from Cell Signaling Technology (Danvers, MA, USA) and BD Biosciences (San Jose, CA, USA), respectively. The antibody against β-actin (A1978, clone AC-15) was purchased from Sigma-Aldrich (St. Louis, MO, USA).

### 4.2. Rat Implantation Model

The animal care and surgery protocols were approved by the institutional animal care committees of the Tokyo University of Pharmacy and Life Sciences, and were performed in compliance with institutional guidelines for experimental animal care (#P1467, #P1563). Wistar-Imamichi rats (Imamichi Institute for Animal Reproduction, Ibaraki, Japan) were kept at constant temperature (24 °C) and humidity (55 ± 5%) with free access to food and water. Eight-week-old female rats were mated at proestrus with 10-week-old males of the same strain. Day 1 of pregnancy was determined by the presence of a vaginal plug or sperm. Complete uteri were collected from days 3 to 5, and the implantation sites on days 7 and 9 were separated from the inter-implantation sites by dissection.

Delayed implantation was induced by ovariectomy on day 4, as described previously [30]. Pregnancy was maintained by daily subcutaneous (sc) injection of P4 (3 mg/rat in sesame oil). Six days after the surgery, each rat was given 0.5 μg of E2 sc to induce implantation, and the uteri were isolated 48 h later.

To determine the effects of E2 and P4 on uterine CALR levels, animals were ovariectomized 14 days before a single administration of E2 (0.5 μg, sc) and/or P4 (3 mg, sc). The uteri were collected 24 h after injection of the steroids.

Pseudopregnancy was induced by mating females with vasectomized males of the same strain. On day 5 of pseudopregnancy, sesame oil (100 μL) was infused into one uterine horn to induce decidualization. The non-infused horn was used as a control.

Each tissue was fixed immediately in 4% paraformaldehyde for immunohistochemical analysis or pooled for immunoblotting and RNA extraction.

### 4.3. Immunohistochemistry

Paraffin sections of 4% paraformaldehyde-fixed uteri were deparaffinized and rehydrated. After boiling for 20 min in 10 mM citrate buffer (pH 6.0), the sections were incubated with 3% H_2_O_2_ for 30 min, blocked with 10% normal goat serum, and then incubated with a rabbit polyclonal anti-CALR antibody (1:100), or normal rabbit IgG (1:100, sc-2027; Santa Cruz Biotechnology, Dallas, TX, USA) as a negative control, overnight at 4°C. Subsequently, the sections were incubated with Histofine Simple Stain MAX-PO MULTI (Nichirei Biosciences, Inc., Tokyo, Japan) and then with DAB. The sections were counterstained with methyl green.

### 4.4. Immunoblotting

Uterine tissues were homogenized with a Polytron in an ice-cold lysis buffer (50 mM Tris-HCl buffer (pH 7.5) containing 0.15 M NaCl, 10 mM EDTA, 0.1% Tween-20, 5 μg/mL aprotinin, 5 μg/mL leupeptin, 0.1 mM phenylmethylsuffonylfluoride, and 0.1% β-mercaptoethanol). Protein lysates from ESCs were prepared with an RIPA buffer (Cell Signaling Technology) according to the manufacturer’s instructions. The homogenate was centrifuged at 13,000× *g* to remove insoluble protein and obtain the crude supernatants. The protein concentration was determined using Bio-Rad protein dye reagent (Bio-Rad Laboratories Inc., Hercules, CA, USA). Equal amounts of protein (20 μg) were separated by 5–20% gradient SDS-PAGE (SuperSep Ace, Fujifilm Wako Pure Chemical Corp., Osaka, Japan) and then electrophoretically transferred to polyvinylidene difluoride membranes (Merck Millipore, Burlington, MA, USA). The membranes were probed with primary antibodies against CALR (1:2500) and Cx43 (1:2500). Immunoreactive bands were detected using enhanced chemiluminescence (Merck Millipore) after incubation with horseradish peroxidase-labeled goat, anti-mouse, or anti-rabbit IgG (0.5 μg/mL, Vector Laboratories, Burlingame, CA, USA). Membranes were then treated with stripping solution (25 mM glycine-HCl, pH 2.0, containing 1% (*w/v*) SDS) and re-probed with an antibody against GAPDH (0.2 μg/mL). Relative band intensity was assessed by means of a densitometry analysis of digitalized autographic images using ImageJ software (NIH) and normalized to that of GAPDH.

### 4.5. Preparation of Rat ESCs and Treatment with siRNAs

Uteri were removed from day 5 pregnant rats. Uterine cells, consisting mainly of ESCs, were prepared by a modified method of enzymatic dissociation [31]. In brief, the uterine tissues were slit longitudinally and then minced and digested with Ca^2+^- and Mg^2+^-free balanced PBS containing dispase (6 mg/mL) and pancreatin (25 mg/mL). After repeated pipetting for 10 s using a 25 mL pipette, the supernatant containing luminal epithelial cells was discarded. ESCs were isolated by digestion at 37 °C for 60 min with collagenase (0.5 mg/mL, type I, Sigma-Aldrich, St. Louis, MO, USA)). The purity of stromal cells collected was greater than 95%, as determined by immunocytochemical detection of the stromal marker vimentin and the epithelial marker cytokeratin. Subsequently, the cell suspension was washed, filtered through a cell strainer (70 μm) to remove clumps, and transferred to a 24-well culture dish (3 × 104 cells/well) in Dulbecco’s modified Eagle medium supplemented with 10% fetal bovine serum. The cells were cultured in Dulbecco’s modified Eagle medium and Ham’s F-12 (DMEM/F12; Invitrogen, Carlsbad, CA, USA) supplemented with 10% (*w/v*) charcoal-stripped fetal bovine serum, 50 μg/mL penicillin/streptomycin, 100 μg/mL neomycin, and 0.5 μg/mL amphotericin B. The medium was changed 1 h later to remove unattached cells, including epithelial cells. To determine the impact of silencing CALR expression on decidualization, ESCs were transfected with a CALR-specific siRNA (30 pmol/well; 5′-GGA UAA AGG GUU GCA GAC AAG CCA A-3′, 5′-UUG GCU UGU CUG CAA CCC UUU AUC C-3′, Invitrogen) or a control siRNA (30 pmol/well; Qiagen, Mississauga, ON, Canada) for 24 h, and then treated for 48 h with MPA (100 nM) and db-cAMP (0.5 mM) for decidual stimulation.

### 4.6. Preparation of Endometrial Samples from Infertile Patients and Ovarian Steroid Assay

Endometrial tissues and blood samples were collected from 27 patients aged 26–43 years with endometrial polyps. All patients had taken no hormonal medications for 3 months before surgery and had regular menstrual cycles. In addition, with the exception of polyps, there were no known reasons underlying the patients’ infertility. The menstrual cycle stage of each subject was determined based on menstrual history and serum E2 and P4 levels at the time of sample collection. The serum levels of E2 and P4 were measured by electrochemiluminescence immunoassay at the SRL Shinjuku laboratory (Tokyo, Japan). Endometrial tissues from the normal areas of the endometrium were obtained during surgery, just before resection of the uterine polyps by transcervical resectoscopy. Tissues were collected in the secretory phase of the menstrual cycle. These pre-polypectomy samples were frozen immediately and stored at −80 °C until RNA extraction. One to two months later, post-polypectomy samples were collected from the same patients by endometrial suction during the secretory phase (day 15–25). All subjects were enrolled in the study between 2013 and 2014.

### 4.7. RNA Isolation and Quantitative Real-Time RT-PCR

RNA was extracted using an Isogen reagent (Nippon Gene, Tokyo Japan) according to the manufacturer’s instructions. The expression levels of the mRNAs encoding the rat decidual markers Prl, Dtprp, and Cx43, as well as those of the human pregnancy-related proteins *IGFBP1*, *IGFBP7*, *PTGS2*, and *CALR*, were measured by quantitative real-time RT-PCR using the iScript™ One-Step RT-PCR kit with SYBR^®^ Green (Bio-Rad Laboratories). The primers used are listed in Table 1. The fold change in the expression of each gene was calculated using the ΔΔCt method with glyceraldehyde 3-phosphate dehydrogenase (*GAPDH*) as an internal control [31].

### 4.8. Statistical Analysis

Data are expressed as the mean ± SEM. Statistical significance was assessed using Student’s *t*-test or one-way analysis of variance (ANOVA) followed by a Tukey–Kramer multiple comparisons test. *p* < 0.05 was considered statistically significant.

## Figures and Tables

**Table 1:** Primers for real-time PCR analyses.

Name. (Accession No.)	Sequence (5′–3′)	Product Length (bp)
Rat *Prl*	CATGCTTCTCACTACATCCAT	133
(NM_012629.1)	CTTCAGGAGTAGCTAGGGAAGA	
Rat *Dtprp*	ATCCAGCGAGCTGAAGTCAT	114
(NM_022846.2)	ATGCCTATACATGCGTGCAA	
Rat *CALR*	GACCTCTGGCAGGTCAAGTC	71
(NM_022399.2)	TCAGCGTATGCCTCATCGT	
Rat *Cx43*	CGAAAACGTCTGCTATGACAAG	112
(NM_012567.2)	ATAGAACACATGGGCCAAGTAC	
Rat *GAPDH*	AAAGCTGTGGCGTGAGG	96
(NM_017008.4)	TTCAGCTCTGGGATGACCTT	
Human *IGFBP-1*	AATGGATTTTATCACAGCAGACAG	73
(NM_000596.4)	GGTAGACGCACCAGCAGAGT	
Human *IGFBP-7*	AAGTAACTGGCTGGGTGCTG	86
(NM_001553.3)	CTGTCCTTGGGAATTGGATG	
Human *PTGS2*	CTTCACGCATCAGTTTTTCAAG	96
(NM_000963.4)	TCACCGTAAATATGATTTAAGTCCAC	
Human *CALR*	GACCTCTGGCAGGTCAAGTC	71
(NM_004343.4)	TCAGCGTATGCCTCATCGT	
Human *GAPDH*	AGCCACATCGCTCAGACA	66
(NM_002046.7)	GCCCAATACGACCAAATCC	
